# Autologous hematopoietic transplantation following COVID‐19 infection

**DOI:** 10.1002/ccr3.3712

**Published:** 2021-01-03

**Authors:** Marthilde Brzycki, Robert Richard, Nicholas Burwick, Solomon Graf, Craig O'Brien, Daniel Wu, Thomas R. Chauncey

**Affiliations:** ^1^ Marrow Transplant Unit VA Puget Sound Health Care System Seattle WA USA; ^2^ Division of Hematology Department of Medicine University of Washington Seattle WA USA; ^3^ Division of Medical Oncology Department of Medicine University of Washington Seattle WA USA; ^4^ Clinical Research Division Fred Hutchinson Cancer Research Center Seattle WA USA

**Keywords:** autologous transplantation, COVID‐19, multiple myeloma

## Abstract

Autologous hematopoietic cell transplantation following induction therapy is standard of care for most patients with newly diagnosed multiple myeloma (*N Engl J Med* 2017, **376**; 1311). Though active COVID‐19 infection is typically a contraindication to aggressive therapy, little is known about the safety of autologous transplantation after resolution of acute symptoms and undetectable pathogen by nasopharyngeal PCR.

## Graphical abstract

The majority of patients with underlying hematologic malignancy will survive acute COVID‐19 infection. For many, subsequent dose‐intensive therapy with autologous transplantation will be critical for optimal outcomes, and those with clinical recovery from COVID‐19 infection should not be precluded from consideration of autologous hematopoietic cell transplantation.

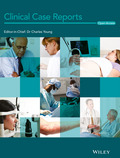

## 1 CASE REPORT

Autologous hematopoietic cell transplantation following induction therapy is standard of care for most patients with newly diagnosed multiple myeloma.^1^ Though active coronavirus disease‐2019 (COVID‐19) infection is typically a contraindication to aggressive therapy, little is known about the safety of autologous hematopoietic cell transplantation (HCT) after apparent resolution of acute symptoms and undetectable pathogen by nasopharyngeal PCR. We report here a case of newly diagnosed multiple myeloma who developed acute COVID‐19 infection following induction and chemomobilization, experienced protracted viral detection, then underwent autologous HCT with minimal early toxicity. His post‐transplant course and later convalescence were complicated by a number of clinical problems.

A 72‐year‐old man was diagnosed with multiple myeloma, lambda light chain subtype, Revised‐International Staging System‐2 (R‐ISS‐2), after presenting with fatigue and weight loss, and was found to have anemia, thrombocytopenia, renal insufficiency, proteinuria, hypercalcemia, and multiple lytic bone lesions. Marrow showed 45% abnormal plasma cells that were lambda light chain restricted and serum lambda free light chain was elevated to 15.450 gm/L. Fluorescence in situ hybridization (FISH) analysis showed gain of CKS1B at 1q21.3, monosomy 13, and loss of IgH at 14q32. He received a course of plasmapheresis along with 4 cycles of cyclophosphamide‐bortezomib‐dexamethasone (CyBorD) therapy with complete response as assessed by normalization of serum free light chains and marrow plasma cell percentage.

Considering the high‐risk profile of R‐ISS‐2, markedly elevated light chains, and amplification of 1q on FISH,^2^ along with complete resolution of COVID‐19 symptoms and return to baseline performance status, he proceeded to autologous HCT.^1^ He underwent chemomobilization with cyclophosphamide (2 gm/m^2^) and dexamethasone followed by high‐dose granulocyte‐colony stimulating factor (G‐CSF) and collected 4.5 × 10e6 CD34/kg on day 11 after chemotherapy. The evening of collection, he developed fever to 101°F, nonproductive cough, sore throat, diarrhea, and increasing fatigue. Nasopharyngeal swab was PCR positive for COVID‐19 (CEPHEID^®^), and chest radiography was unremarkable. Symptoms resolved within 3 days, and he went on to experience intermittent fever to 99°F, though performance status eventually improved to baseline. He continued to test positive for COVID‐19 by PCR on days 28 and 46 after the initial positive test, though quantitative results or cycle thresholds were not available.

After three negative COVID‐19 tests on days 67, 71, and 88 after the initial positive test, he received high‐dose melphalan (200 mg/m^2^) followed by autologous peripheral blood stem cell (PBSC) infusion. He engrafted with sustained neutrophil recovery and platelet transfusion‐dependence on day 13 without unexpected toxicities. He became independent in activities of daily living (ADL) with stable weight and normal nutritional and fluid intake.

On day 29 after transplantation, he developed moderate somnolence and increased fatigue that interfered with nutrition and fluid intake, ultimately requiring hospital admission. The next day he developed acute delirium with confusion, impulsivity, and forgetfulness, lasting for approximately 1 week. Metabolic and infectious evaluations were unremarkable. On day 32 after transplantation, he developed bilateral lower extremity weakness requiring a wheelchair as well as an increase in baseline hearing impairment confirmed by audiometry evaluation. Neurologic examination was otherwise unremarkable, and MRI brain was without abnormalities. COVID‐19 antibody testing (Abbott SARS‐CoV‐2 IgG EUA Assay) was nonreactive 128 days and 178 days after initial COVID‐19 documentation. Though lower extremity weakness remains, with ongoing physical therapy he has slow improvement and is now able to climb stairs and ambulate with walker assistance, while hearing has improved to baseline.

## 2 DISCUSSION

The effects of COVID‐19 infection in patients with multiple myeloma and other hematologic malignancies^3^, ^4^, ^5^, ^6^, ^7^, ^8^, ^9^ are confirmed to show increased mortality, particularly for those with older age, poorly controlled malignancy, decreased performance status, hospitalization, as well as prior autologous or allogeneic HCT. We describe here the clinical course of autologous transplantation following COVID‐19 infection. This case illustrates several important issues that will require validation and exploration as prospective clinical information becomes available.

Dose‐intensive therapy with autologous PBSC rescue following symptomatic COVID‐19 infection can be accomplished without evidence of recurrent excretion or respiratory symptoms. Whether this single report of autologous transplantation after COVID‐19 infection represents a unique occurrence or is consistent with more general expectations remains to be determined. The relatively lower intensity of high‐dose melphalan monotherapy in contrast to other preparative regimens for HCT, as well as other patient‐specific clinical factors, should be considered in this assessment.

Resolution of COVID‐19 detection was not accompanied by demonstrable seroconversion as has been observed in other settings.^10^, ^11^ Whether this can be attributed to the limits of antibody detection, the underlying myeloma physiology,^12^, ^13^ cytotoxic chemotherapy, the severity of the infection, and/or a preferential cellular immunity response is unknown. Though the kinetics of immune response is not well defined,^14^ limited data suggest that IgG seroconversion can approach 80% following acute COVID‐19 infection in immunocompetent patients.^15^ It could be speculated that recovery from initial infection despite protracted shedding in the absence of serologic response was consistent with an effective cellular immune response.

COVID‐19 infection in patients with malignancy, whether or not receiving active therapy, can be associated with protracted viral detection in the absence of clinical symptoms.^16^, ^17^ Whether positive PCR testing following acute infection represents active virus or shedding of replication‐incompetent viral particles is an issue that current routine clinical testing is not empowered to resolve.

Whether pretransplant COVID‐19 infection contributed to this patient's post‐transplant morbidities or whether these symptoms were within the expected spectrum seen during post‐transplant recovery will require further clarification and experience. Whether this patient's relatively milder symptoms during the acute COVID‐19 infection or the time interval from acute infection to autologous transplantation influenced outcomes are also considerations. His later symptoms of transient delirium, exacerbation of underlying hearing impairment, and delayed‐onset lower extremity weakness appear unusual for what is typically seen in those with similar disease and treatment profiles in the absence of prior COVID‐19 infection, though have been reported following COVID‐19 infection in the nontransplant setting.^18^, ^19^, ^20^, ^21^ He is currently over 100 days from transplantation and has not yet recovered to his pretransplant performance status.

This description of one patient's experience with autologous transplantation following COVID‐19 infection does not predict how a diverse group of patients would fare but rather speaks to the need to establish databases for population‐based assessment. While there has been no evidence of COVID‐19 reactivation during his transplant course, a protracted functional recovery following transplantation was observed and should be considered in transplant decisions as well as assessment and management of similar patients along with standard clinical care.

Autologous PBSC transplantation for multiple myeloma was pursued following COVID‐19 infection without evidence of reactivation of infection. Serology response may be limited, and long‐term effects of prior COVID‐19 infection may further affect post‐transplant recovery in some patients. As increasing numbers of patients with malignancy survive COVID‐19 infection, incidence and severity of attributable morbidities after subsequent therapies will require further evaluation and care.

## CONFLICT OF INTEREST

The authors declare no competing financial interests. No research funding was associated with this report. The subject provided informed consent for treatment and correlative research.

## AUTHOR CONTRIBUTIONS

MB and TRC: involved in conception and design, and primary preparation/writing of manuscript. MB, RR, NB, SG, CO, DW, and TRC: reviewed, edited, and approved the manuscript.
